# Black Hairy Tongue Observed During Esophagogastroduodenoscopy

**DOI:** 10.7759/cureus.74331

**Published:** 2024-11-23

**Authors:** Masaya Iwamuro, Takehiro Tanaka, Motoyuki Otsuka

**Affiliations:** 1 Department of Gastroenterology and Hepatology, Okayama University Graduate School of Medicine, Dentistry, and Pharmaceutical Sciences, Okayama, JPN; 2 Department of Pathology, Okayama University Hospital, Okayama, JPN

**Keywords:** candidiasis, esophagogastroduodenoscopy, fungal infection, histopathology, pigmented lesions

## Abstract

Black hairy tongue, also known as *lingua villosa nigra*, is a benign oral condition characterized by a dark discoloration and “hairy” appearance on the tongue’s dorsal surface, resulting from elongated filiform papillae. This condition is associated with risk factors such as smoking, poor oral hygiene, and diabetes, which increase susceptibility to microbial colonization, particularly by *Candida* species. Although commonly diagnosed by visual inspection, black hairy tongue is infrequently observed during endoscopic procedures. We report a case of a 69-year-old Japanese man with poorly controlled type 2 diabetes (hemoglobin A1c of 9.7%) and a significant smoking history of 49 pack-years. During a routine esophagogastroduodenoscopy, a dark lesion was detected on the dorsal surface of the tongue. Detailed imaging and biopsy revealed elongated papillae with fungal hyphae, confirming a diagnosis of candidiasis. This case underscores the value of esophagogastroduodenoscopy and histopathological examination in diagnosing black hairy tongue and distinguishing it from other pigmented lesions. Recognizing black hairy tongue as a potential finding during endoscopy may aid in prompt diagnosis, especially in patients with predisposing factors like smoking and diabetes.

## Introduction

Black hairy tongue (*lingua villosa nigra*) is a benign condition characterized by the black or brown discoloration of the tongue's dorsal surface due to the elongation of the filiform papillae. This elongation allows the papillae to trap debris, bacteria, and fungi, giving the tongue a dark, "hairy" appearance. Common risk factors include smoking, poor oral hygiene, and conditions such as diabetes that predispose individuals to fungal colonization, particularly with *Candida* species [1-5]. Although typically diagnosed via visual inspection, endoscopic observation of a black hairy tongue is uncommon.

This report describes a unique case of black hairy tongue diagnosed during esophagogastroduodenoscopy with histopathological confirmation of coexisting candidiasis, underscoring the potential diagnostic role of endoscopy in identifying and distinguishing this condition from other pigmented lesions.

## Case presentation

A 69-year-old Japanese man presented for routine esophagogastroduodenoscopy as part of a screening examination. His medical history included type 2 diabetes, poorly controlled despite sitagliptin treatment, with a hemoglobin A1c level of 9.7%. He also had a significant smoking history of 49 pack-years and reported consuming approximately 30 grams of alcohol daily. The patient did not have any symptoms related to the oropharynx or tongue.

**Figure 1:**
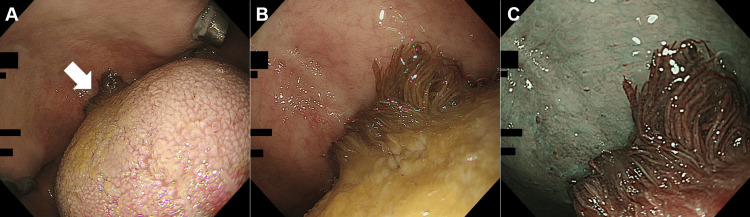
Endoscopic images of black hairy tongue. (A) Endoscopic view of the tongue showing a black, protruding lesion (arrow) on the dorsal surface during esophagogastroduodenoscopy. (B) Close-up view under white light revealing elongated, dark papillae. (C) Narrow-band imaging further highlights the structure of the elongated papillae and the dark coloration.

**Figure 2:**
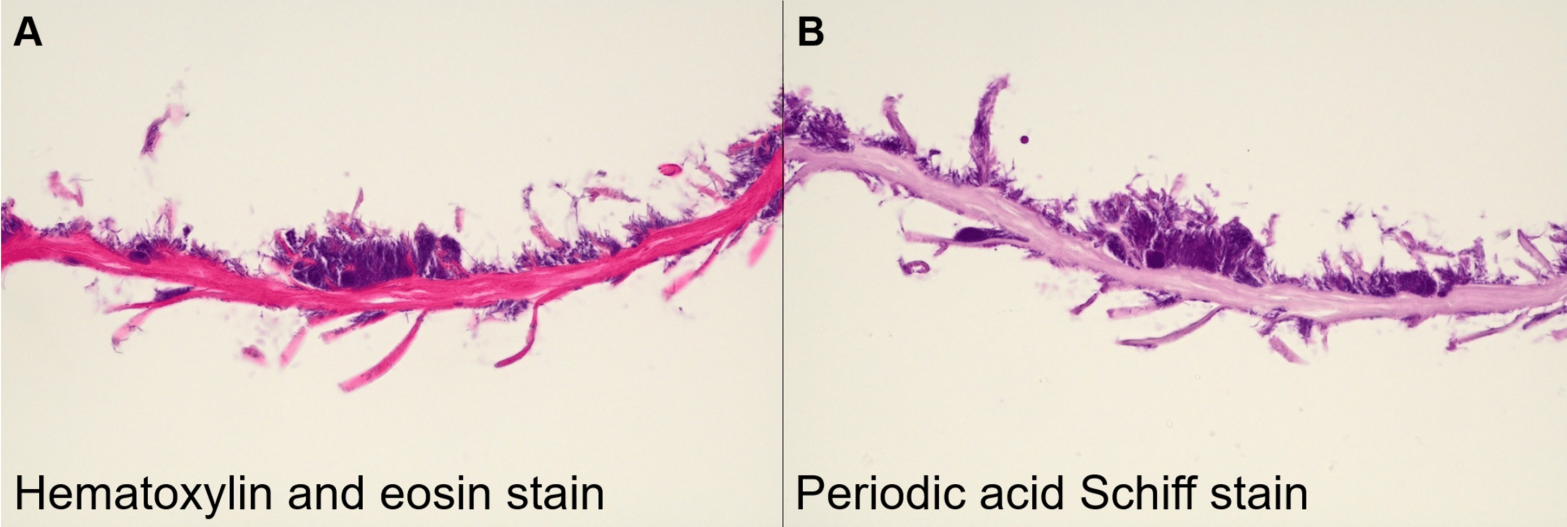
Histopathological findings of the biopsy specimen. (A) Hematoxylin and eosin (H&E) staining showing elongated papillae with numerous adherent hyphae, consistent with candidiasis. (B) Periodic acid-Schiff (PAS) staining confirming the presence of fungal hyphae, further supporting the diagnosis of candidiasis.

During esophagogastroduodenoscopy, a black, hairy protrusion was noted on the dorsal surface of the tongue (Figure 1A, arrow). Close-up imaging under white light (Figure 1B) and narrow-band imaging (Figure 1C) revealed prominent papillae with a characteristic dark color. Endoscopic biopsy specimens demonstrated elongated papillae with numerous adherent hyphae, confirmed by hematoxylin and eosin (H&E) staining (Figure 2A) and periodic acid-Schiff (PAS) staining (Figure 2B), supporting a diagnosis of candidiasis. The patient was advised to cease smoking, maintain rigorous oral hygiene, and pursue better diabetes management to prevent recurrence.

## Discussion

Black hairy tongue is an uncommon, generally benign condition, resulting from excessive growth and delayed desquamation of the filiform papillae. When not regularly shed, these elongated papillae accumulate substances such as bacteria, yeast, food particles, and pigments from tobacco or coffee, resulting in the characteristic dark or "hairy" appearance [1-5]. Candidiasis, as noted in this case, is a common underlying factor, particularly in patients with diabetes, poor oral hygiene, or compromised immune function.

In this case, esophagogastroduodenoscopy was instrumental in detecting black hairy tongue, an unusual diagnostic approach for this condition. PubMed searches using the terms "black tongue" and "endoscopy" or "black tongue" and "esophagogastroduodenoscopy" yielded only one article authored by our group [6], underscoring the rarity of endoscopic diagnosis for this condition. Though primarily diagnosed by visual examination, endoscopy can provide detailed visualization and facilitate biopsy when needed, aiding in the differential diagnosis of other pigmented oral lesions, including malignant melanoma. Given that melanoma can also appear darkly pigmented, distinguishing these conditions is essential [7-9]. Histopathological findings - such as the presence of hyphae, elongated papillae, and positive PAS staining - help confirm candidiasis rather than a neoplastic process.

Treatment for black hairy tongue generally involves improving oral hygiene, cessation of smoking, and addressing any predisposing factors, such as poorly controlled diabetes [10-12]. This patient was counseled on these lifestyle modifications to support recovery and prevent recurrence.

## Conclusions

This case highlights the utility of endoscopy in diagnosing black hairy tongue, particularly when coupled with histopathological examination to rule out other pigmented lesions. Endoscopists should be aware of black hairy tongue as a potential finding and recognize its benign nature, provided that thorough evaluation excludes malignancy.
